# STAT3 inhibitor mitigates cerebral amyloid angiopathy and parenchymal amyloid plaques while improving cognitive functions and brain networks

**DOI:** 10.1186/s40478-021-01293-5

**Published:** 2021-12-15

**Authors:** Jogender Mehla, Itender Singh, Deepti Diwan, James W. Nelson, Molly Lawrence, Eunjae Lee, Adam Q. Bauer, David M. Holtzman, Gregory J. Zipfel

**Affiliations:** 1 Department of Neurological Surgery, Washington University School of Medicine, St. Louis, MO 63110 USA; 2 Hope Center for Neurologic Disease, Washington University School of Medicine, St. Louis, MO 63110 USA; 3 Charles F. and Joanne Knight Alzheimer’s Disease Research Center, Washington University School of Medicine, St. Louis, MO 63110 USA; 4 Department of Neurology, Washington University School of Medicine, St. Louis, MO 63110 USA; 5 Department of Radiology, Washington University School of Medicine, St. Louis, MO 63110 USA; 6 Department of Biomedical Engineering, Washington University School of Medicine, St. Louis, MO 63110 USA

**Keywords:** Alzheimer’s disease, Cerebral amyloid angiopathy, STAT3, Amyloid-β, Reactive oxygen species, Neuroinflammation, Functional connectivity

## Abstract

Previous reports indicate a potential role for signal transducer and activator of transcription 3 (STAT3) in amyloid-β (Aβ) processing and neuritic plaque pathogenesis. In the present study, the impact of STAT3 inhibition on cognition, cerebrovascular function, amyloid pathology, oxidative stress, and neuroinflammation was studied using in vitro and in vivo models of Alzheimer’s disease (AD)-related pathology. For in vitro experiments, human brain vascular smooth muscle cells (HBVSMC) and human brain microvascular endothelial cells (HBMEC) were used, and these cultured cells were exposed to Aβ peptides followed by measurement of activated forms of STAT3 expression and reactive oxygen species (ROS) generation. Further, 6 months old 5XFAD/APOE4 (5XE4) mice and age-matched negative littermates were used for in vivo experiments. These mice were treated with STAT3 specific inhibitor, LLL-12 for 2 months followed by neurobehavioral and histopathological assessment. In vitro experiments showed exposure of cerebrovascular cells to Aβ peptides upregulated activated forms of STAT3 and produced STAT3-mediated vascular oxidative stress. 5XE4 mice treated with the STAT3-specific inhibitor (LLL-12) improved cognitive functions and functional connectivity and augmented cerebral blood flow. These functional improvements were associated with a reduction in neuritic plaques, cerebral amyloid angiopathy (CAA), oxidative stress, and neuroinflammation. Reduction in amyloid precursor protein (APP) processing and attenuation of oxidative modification of lipoprotein receptor related protein-1 (LRP-1) were identified as potential underlying mechanisms. These results demonstrate the broad impact of STAT3 on cognitive functions, parenchymal and vascular amyloid pathology and highlight the therapeutic potential of STAT3 specific inhibition for treatment of AD and CAA.

## Introduction

Alzheimer’s disease (AD) is a multifactorial chronic devastating neurodegenerative disease that is the most common cause of dementia in the elderly [1, 2]. Pathologically, AD is characterized by deposition of extracellular parenchymal amyloid plaques and intracellular formation of neurofibrillary tangles that are responsible for various cascades including oxidative stress, neuroinflammation, neuronal cell death, cholinergic deficit, synaptic dysfunction, and functional connectivity (FC) deficits that ultimately lead to cognitive impairment [1, 3]. Cerebral amyloid angiopathy (CAA) is characterized by deposition of amyloid-β (Aβ) peptides in pial and penetrating cerebral blood vessels leading to vascular oxidative stress, vascular inflammation, impaired Aβ clearance across the blood–brain-barrier, and deficits in cerebral perfusion [4]. CAA is almost universally identified in AD patients [5, 6], and CAA is also found in elderly patients without classic AD pathology. In both circumstances, CAA has been causally linked to progressive cognitive decline. To date, no drug therapy has been shown to stop the progression of AD or CAA, and current therapies are primarily directed towards providing temporary relief of initial cognitive symptoms.

Signal transducer and activator of transcription 3 (STAT3), belonging to the STAT family, is a transcription factor that is present in neurons, blood vessel endothelial, astrocytes and microglia [7]. STAT3 is activated through phosphorylation by Janus kinases (JAK) in response to cytokines, intercellular mediators, and growth factors that result in various biological outcomes such as cell proliferation, differentiation, and apoptosis [8, 9]. Acetylation is another post-translational modification that has been reported to augment STAT3 function by enhancing DNA binding, transactivation activity and nuclear localization [10]. These activated forms of STAT3 have been shown to be upregulated in brains of APP/PS1 mice and AD patients, but their role in AD pathophysiology has only recently been evaluated and their role in CAA pathophysiology has yet to be explored.

One of the most important and best studied roles of STAT3 relates to its promotion of gliosis in a variety of disease states including AD. Activation of the JAK/STAT3 signaling pathway has been strongly correlated to the presence of reactive astrocytes in animal models of AD [11, 12] as well as stroke [13] and epilepsy [14]. Ramified and activated microglia have been shown to enhance astrogliosis through a STAT3-mediated mechanism [15, 16]. Inhibition of the astrocyte-specific JAK/STAT3 pathway via genetic overexpression of suppressor of cytokine signaling 3 (SOCS3) was shown to prevent astrocyte reactivity and decrease microglial activation in AD animal models [11]. More recently, the functional consequences of STAT3-mediated glia activation in AD have begun to be explored. Chen and colleagues found in vitro that STAT3 inhibition via siRNA treatment decreased IL-1β and TNF-α-induced astrocyte differentiation and partially restored neuronal differentiation [17]. Reichenbach and colleagues showed in vivo that astrocyte‐specific deletion of STAT3 reduced pro‐inflammatory cytokine activation, decreased dystrophic neurite burden, and reduced neuritic plaque load leading to improved spatial learning and memory in aged APP/PS1 mice [12].

Whether STAT3 plays a causal role in other pathophysiological elements of AD including functional connectivity deficits, vascular oxidative stress, CAA load, and cerebral autoregulatory dysfunction has yet to be examined. The downstream molecular events by which STAT3 contributes to the pathophysiology of AD and CAA are also not known. In the present study, we performed a complementary set of experiments focused on both parenchymal and vascular components of AD. We utilized both in vitro and in vivo experimental models. In the former, STAT3 inhibition was achieved both genetically and pharmacologically. In the latter, STAT3 inhibition was achieved via administration of the STAT3-specific inhibitor, LLL-12. Finally, we explored several potential molecular mechanisms by which STAT3 inhibition imparts its beneficial effects on AD and CAA-related pathologies.

## Materials and methods

### Cell culture experiments

#### Reagents

We purchased Aβ peptides (Aβ1-40 and Aβ1-42) from American Peptide Company, Sunnyvale, CA. Aβ peptides were reconstituted in purified water, snap frozen in liquid nitrogen and immediately used or stored at − 80 °C for no longer than 2 weeks prior to use. STAT3 inhibitors, LLL-12 (cat.- 573131) and S31-201 (cat.- SML0330) were purchased from MilliporeSigma, USA. LLL-12 is also known as 5-hydroxy-9,10-dioxo-9,10-dihydroanthracene-1-sulfonamide (molecular formula- C_14_H_9_NO_5_S). The chemical name for S31-201 is 2-Hydroxy-4-[[[[(4-methylphenyl)sulfonyl]oxy]acetyl]amino]-benzoic acid (molecular formula- C_16_H_15_NO_7_S). These should be stored at − 20 °C for long-term use. These are soluble in dimethyl sulfoxide (DMSO). Human brain vascular smooth muscle cells, human brain microvascular endothelial cells and cells medium were purchased from ScienCell Research Laboratories.

#### Cell culture lines

Human brain vascular smooth muscle cells (HBVSMC) and human brain microvascular endothelial cells (HBMEC) were grown as per manufacture’s instruction using smooth muscle cells (SMCM, Cat. #1101) and endothelial cells (ECM, 1001) complete medium. HBVSMC and HBMEC were used at passages 4 and maintained at 37 °C in humidified air containing 5% CO_2_.

#### Reactive oxygen species (ROS) assay

We performed ROS assay as described previously [18]. Briefly, we used 96-well black clear-bottomed plates and in each well 5.0 × 10^4^ cells were placed the day prior to experiments. We started the experiments when cell density was near to 90–95% confluency, later, cells were washed slowly for 10–15 s with Leibovitz’s media (L-15; no phenol red indicator, at 37 °C) and then, loaded with Mitotracker Red CM-H2XRos (MTR, ROS-sensitive dye, final concentration of 5 μM) or dihydroethidium (DHE, final concentration of 10 μM). Then, plates were incubated for 20–30 min. After incubation, cells were washed with warm L-15 media. Then, Aβ peptides (1 µM) were prepared freshly in L-15 media and added immediately just before fluorescence measurements. In some experiments, cells were pretreated with STAT3 inhibitors (LLL-12 at 5 μM or S31-201 at 10 μM or siRNA for STAT3) prior to Aβ peptides (1 μM) exposure. Then, we measured the fluorescence at room temperature with a microplate reader (Synergy HTTR using KC4 software) over 30 min (λEx = 475 and λEm = 645 for MTR, and λEx = 520 and λEm = 610 for DHE).

#### STAT3 expression in cultured cells exposed to Aβ peptides

Human brain vascular smooth muscle cells (HBVSMC) and human brain microvascular endothelial cells (HBMEC) were incubated with Aβ1-42 (1 µM) and Aβ1-40 (1 µM) for 24 h, respectively. After 24 h, cells were washed slowly for 15–20 s with cell media and then, cells were collected. Then, cells were lysed with lysis buffer and centrifuged to collect the supernatant. The protein concentration was determined in supernatant using BCA protein assay kit.

An equal amount of protein was subjected to SDS polyacrylamide gel electrophoresis (SDS-PAGE) and later, transferred to polyvinylidene difluoride (PVDF) membrane. After blocking, the membranes were incubated with primary antibodies [rabbit anti-STAT3 from Cell Signaling Technology, 1:1000; rabbit anti- Phospho-STAT3 (Tyr705) from Cell Signaling Technology, 1:1000; rabbit anti-Ac-Lys685-STAT3 (acetylated STAT3) from Cell Signaling Technology, 1:1000; mouse anti-β-tubulin, 1:1000 from Cell Signaling Technology] overnight at 4˚C. After 24 h incubation, membranes were washed with Tris buffered saline with Tween (TBST). Then, the membranes were incubated with secondary antibodies (anti-rabbit-HRP, 1:3000 from Cell Signaling Technology; anti-mouse-HRP, 1:3000 from Cell Signaling Technology) at room temperature for 2 h. Protein bands were visualized using ECL Western blotting detection reagents (GE Healthcare). The density of the target protein was quantified using ImageJ program. Then, target protein density was normalized to β-tubulin density.

### Postmortem experiment in brains of AD patients

#### Isolation of cerebral microvessels

Frozen postmortem brain samples from AD patients were provided by the Charles F. and Joanne Knight Alzheimer Disease Research Center (Knight ADRC) at Washington University in St Louis. Cerebral microvessels from the postmortem AD brain samples were isolated as previously described [19]. Briefly, these tissues were homogenized in 3.5-fold excess volume of ice-cold buffer solution [NaCl (130 mM), KCl (4.7 mM), CaCl2 (2.5 mM), KH2PO4 (1.2 mM), MgSO4 (1.2 mM), Hepes (15 mM), NaHCO3 (25 mM), glucose (10 mM), and sodium pyruvate (1 mM)] at pH 7.4, in a 5-mL hand-held glass dounce tissue grinder. An equal volume of 26% dextran (molecular weight 64,000–76,000) was then added to the homogenate and spun at 5800 g for 15 min. The pellet containing the cerebral microvessels was, then, carefully separated from the supernatant. The resuspended pellet in ice-cold buffered solution was filtered using a 100-μm filter, and the filtrate was passed through a 40-μm filter. The microvessels were washed through using ice-cold buffered solution. The filter was reversed to collect the microvessels using ice-cold buffered solution into a low binding tube and centrifuged at 5000 g for 10 min at 4 °C to collect microvessels in pellet form. The supernatant was discarded and the cerebral microvessels were then resuspended in ice-cold lysis buffer, sonicated, and centrifuged at 20,000×g for 20 min, and supernatant was used for protein and Western blot analysis.

#### STAT3 expression in cerebral microvessels isolated from brain of AD patients

In western blotting, equal amount of protein from the supernatant was separated using SDS–polyacrylamide gels in Tris/Glycine/SDS buffer, transferred onto PVDF membranes using Tris/Glycine buffer. Then, membranes were blocked with 5% nonfat milk in TBST and incubated with primary antibodies [rabbit anti- Phospho-STAT3 (Tyr705) from Cell Signaling Technology, 1:1000; rabbit anti-Ac-Lys685-STAT3 (acetylated STAT3) from Cell Signaling Technology, 1:1000; mouse anti-β-tubulin, 1:1000 from Cell Signaling Technology] overnight at 4˚C. After washing with TBST, the membranes were incubated with secondary antibodies (anti-rabbit-HRP, 1:3000 from Cell Signaling Technology; anti-mouse-HRP, 1:3000 from Cell Signaling Technology) at room temperature for 2 h. Protein bands were detected by ECL Western blotting detection reagents (GE Healthcare). The density of the target protein was quantified using ImageJ program. Then, target protein density was normalized to β-tubulin density.

## In vivo experiments

### Animals

Male 5XFAD/APOE4 mice (6 months old) were used in the present study. 5XFAD/APOE4-knockin (5XE4) mice were generated by crossing 5XFAD (line Tg7031) transgenic mice with APOE4 mice [20], which were gifted by the laboratory of Dr. David Holtzman. All mice were genotyped by polymerase chain reaction using tail sniping method. In the present study, negative littermates were used as wild-type (WT) control. In each cage, 4 mice were group-housed in a controlled environment (22 °C–25 °C, 50% humidity and a 12-h light:dark cycle) with free access to laboratory diet and water.

## Experiment 1

This experiment was performed to assess the effect of LLL-12 on neurobehavior and pathology of 5XE4 mice. Six-month-old mice were randomly divided into 4 groups of 9–11 mice per group. Wild-type control mice and 5XE4 control mice were intraperitoneally administered vehicle (10% DMSO, and 20% cremophor EL in PBS) for 2 months. Wild-type drug-treated mice and 5XE4 drug-treated mice were intraperitoneally administered the STAT3-specific inhibitor, LLL-12 (5 mg/kg), for 2 months. After the last dose of vehicle or drug, behavioral tests were performed to assess neurobehavioral functions as described below. Lastly, mice were perfused, and brain samples were taken out for further biochemical estimation.

### Neurobehavioral assessment

We performed novel object recognition, Y-maze, and burrowing tests to assess the cognitive functions of 8 months old mice. Each behavioral test was performed on different day. All behavioral tests and analysis were performed in blinded manner.

#### Novel object recognition and location tasks

A novel object location (NOL, assess spatial hippocampus-dependent memory) and a novel object recognition (NOR, assess perirhinal cortex-dependent memory) tests were performed to assess memory functions in mice. NOL and NOR tests were performed as per methods described previously [19, 21]. In both NOR and NOL trials, objects were cleaned with 70% ethanol after every trial. Objects at “standard position” were placed at 27 cm from opposing corners of the arena, while object at “novel position” was placed at about 18 cm from opposing corners of the arena.

#### NOR test

Mice were placed into the arena with two identical sample objects and allowed to explore for 10 min. Mice were then removed, and one of two identical objects was replaced with a novel object. After a 10-min inter-trial period, mice were returned to the arena and object exploration behavior was assessed for 5 min and recorded using a videocamera. Mice were considered to be engaged in object exploration when its head was oriented within 45° of an object and within 4 cm of it. Rearing with the head oriented upward was also be included if at least one forepaw was on the object. Climbing over or sitting on the objects was not included. Then, percent exploratory preference was calculated.

#### NOL test

Mice were placed into the arena with two identical objects and allowed to explore for 10 min. Mice were then removed and one of the objects was placed to a new location. After a 10-min of inter-trial period, mice were returned to the arena and novel location exploration behavior was measured and recorded using a videocamera for 5 min. Then, percent exploratory preference was calculated.

#### Y-Maze

Y-maze test can be used to assess spatial working as well as reference memory in rodents. This test was done as described previously with slight modifications [22]. The Y-maze consists of three identical arms made of PVC plastic joined in the middle to form a “Y” with dimensions 20 cm high, 10 cm wide, and 60 cm long. Mice were placed into one of the arms of the maze (start arm) and allowed to explore two of the arms for 5 min (training trial). The third arm, which remained closed, was randomly chosen in each trial. After a 30-min inter-trial interval, the blocked arm was uncovered, and mice were returned to the start arm and allowed to explore all three arms for 10 min (test trial). Sessions were recorded by a video camera positioned over the arena, and sample and test phases were replayed for analysis. Then, total number of novel arm entries by mice were calculated.

#### Burrowing task

Mice were also tested for hippocampal-dependent burrowing behavior analysis according to previously published methods [23]. Briefly, at the onset of dark cycle mice was individually placed in cages equipped with a burrow made of a 200 mm long and 68 mm diameter plastic tube. A PVC cap was applied to close one end of the tube. The open end of the tube was raised ∼3 cm by a PVC ring. The burrow was filled with 200 g of mouse food pellets, and mice were allowed to burrow for overnight. The weight of the remaining food pellets inside the burrow was determined to obtain a measurement of the amount burrowed.

### Fluorescent staining for CAA and neuritic plaques in mouse brain

After completion of behavioral experiments, fluorescent staining for CAA and neuritic plaques in mice brain was performed as described by Han et al. [24]. Littermate wild-type and 5XE4 mice were transcardially perfused with PBS and 4% paraformaldehyde (PFA) under isoflurane. Brains were taken out and post-fixed for 24 h in 4% PFA at 4 °C, and later, preserved in 30% sucrose at 4 °C. Fixed brains were sectioned coronally (40 μm) using cryostat. Then, brain sections (4 sections/brain) were washed three times with PBS and permeabilized by incubating in PBS containing 0.25% Triton-X100 (PBS-T) at room temperature for 30 min. Brain sections were incubated with PBS-T containing 2 μM methoxy-X34 at room temperature for 30 min. After 30 min of incubation, brain sections were washed with PBS (3 times for 5 min) and one time with 50% ethanol in PBS for 5 min. Then, brain sections were mounted on a microscopic glass slide and cover-slipped with Vectashield mounting media. Then, brain sections were imaged using a Nikon Eclipse ME600 digital video microscopy system (Nikon Instruments Inc., Melville, NY) and MetaMorph imaging software (Molecular Devices, Sunnyvale, CA). Plaques deposited in blood vessels and parenchymal region were quantified using ImageJ software. The plaques quantification was done in blinded manner.

### Brain tissue preparation for biochemical estimation

Frozen brain samples from mice were weighed and homogenized in 5% of tris buffered saline (TBS) containing a cocktail of protease inhibitors. Homogenate was stored as aliquots at − 80 °C for further biochemical estimation. All biochemical parameters were performed in a blinded manner.

#### Measurement of Aβ levels in brain tissues of mice

Aβ1-40 and Aβ1-42 levels were measured from brain tissues according to the procedures as described previously with slight modifications [25]. Brain homogenate was suspended in 2% sodium dodecyl sulphate (SDS) containing protease inhibitors and centrifuged at 100,000×g for 60 min, and the supernatant fraction was collected for the soluble Aβ ELISA assay. The remaining SDS-insoluble pellet was dissolved in 70% formic acid and centrifuged at 100,000×g for 60 min, and the supernatant was collected for the insoluble Aβ ELISA assay. The soluble and insoluble levels of Aβ1-40 and Aβ1-42 in all samples were determined using human Aβ1-40 and Aβ1-42 ELISA kits (ThermoFisher Scientific) according to the manufacturer's instructions. The levels were expressed as pg/ml.

#### Measurement of BACE1 activity

BACE1 activity was measured in brain tissue of mice using Fluorometric Assay Kit (Sigma-Aldrich) as per manufacturer’s instructions. BACE1 activity was expressed as the Relative Fluorescence Units.

#### Measurement of sAPPβ and γ-secretase levels

Soluble APPβ levels were measured in brain tissue of mice using ELISA kit (MyBioSource) as per manufacturer’s instructions. Soluble APPβ levels were expressed as ng/ml. γ-Secretase levels were also quantified in homogenate of mice brain tissue using ELISA kit (Biomatik) as per manufacturer’s instructions. γ-Secretase levels were also expressed as ng/ml.

#### Measurement of TNFα and IL-1β levels

TNFα and IL-1β levels in supernatant from mice brain homogenate were measured using ELISA kit from Invitrogen/ Thermo Fisher Scientific as per manufacturer’s guidelines. TNFα and IL-1β levels were expressed as pg/mg of protein.

#### Measurement of lipid peroxidation

To assess lipid peroxidation, thiobarbituric acid reactive species (TBARS) levels were measured in mice brain homogenate as described previously [26]. Briefly, malondialdehyde (MDA) was used as the standard at final concentrations between 1 and 20 μM. TBARS assay was performed by adding 55 μl of 1.33% TBA and 95 μl of 20% acetic acid (pH 3.5) to 100 μl of the prepared samples or standards. Then, mixture was incubated for 1 h at 95 °C. After incubation, 250 μl of n-butanol and pyridine mixture (15:1 v/v) was added and gently mixed by inverting the tubes. Then, mixture was centrifuged for 10 min at 5000 rpm. After centrifugation, an absorbance was measured at 535 nm. TBARS values were expressed as ng/mg brain tissue.

### Immunostaining for astrocytosis and microgliosis

We sectioned brain coronally at 40 μm using freezing microtome. We analyzed 3 brain sections from each mouse for immunohistochemistry, and analysis was conducted by an experimenter who was blinded to treatment group. Immunostaining for GFAP and IBA-1, a marker for astrocytosis and microgliosis, respectively, were performed as previously described [27]. In brief, sections were fixed on microscopic glass slides. Then, brain sections on the glass slides were washed with TBS, and then, blocked for 2 h in TBS (containing 0.3% Triton-X and 3% goat serum). After blocking, brain sections were incubated in primary antibodies (rabbit anti-GFAP and rabbit anti-Iba1) at room temperature on the shaker for 24 h. After 24 h, three 10-min washes were performed and brain sections were again incubated with secondary antibodies [goat anti-rabbit-alexa-594 (IgG (H + L), 1:1000 for GFAP and IBA-1] for 24 h. Following incubation, three 10-min washes were done. Then, brain sections on the slides were covered with coverslips using Vectashield H-1000 (Vector Laboratory). Later, the slides were sealed with nail polish. Then, brain sections were imaged using a Nikon Eclipse ME600 digital video microscopy system (Nikon Instruments Inc., Melville, NY) and MetaMorph imaging software (Molecular Devices, Sunnyvale, CA). The images were analyzed using Image-J software.

### Western blot for LRP-1 expression

Mice brain were homogenized in lysis buffer supplemented with protease inhibitors cocktail. Then, samples were centrifuged at 10,000×g for 15 min at 4 °C. The supernatant was collected, and protein levels were measured by BCA protein assay kit. For analysis of unmodified LRP-1, equal amount of protein from the supernatant was separated using SDS–polyacrylamide gels in Tris/Glycine/SDS buffer, transferred onto PVDF membranes, and incubated with mouse anti-LRP-1 monoclonal antibody (ThermoFisher Scientific, 1:1000). After washing with TBST, the membranes were incubated with secondary antibody (anti-mouse-HRP, 1:3000 from Cell Signaling Technology) at room temperature for 2 h. Protein bands were detected by ECL Western blotting detection reagents (GE Healthcare). After imaging, blots were stripped and confirmed for absence of signal. Blots were then re-probed with mouse anti-β-actin (abcam, 1:1000) overnight at 4˚C. After washing with TBST, the membranes were incubated with secondary antibody (anti-mouse-HRP) at room temperature for 2 h. Protein bands were visualized by ECL Western blotting detection reagents. The density of the target protein was quantified using ImageJ program. Then, target protein density was normalized to β-actin density.

For analysis of oxidative modification of LRP-1 (HNE4 bound LRP-1), immunoprecipitation of LRP-1 from brain homogenate was performed. Briefly, 150 μg of protein from brain homogenate was incubated with 500 μl immunoprecipitation (IP) buffer, and precleared with 50 μl washed protein A/G sepharose beads (Rockland Immunochemicals, Inc) for 90 min at 4 °C. The precleared extract was then incubated overnight at 4 °C with 10 μg anti-LRP-1 mouse monoclonal antibody (ThermoFisher Scientific) followed by 90-min incubation with protein A/G sepharose beads (50 μl). The antigen–antibody-protein A/G complex was centrifuged at 5000×g for 3 min and the resultant pellet was washed 5 times with IP buffer. The antigen–antibody complex was eluted by adding 25 μl sample buffer for SDS-PAGE and heating at 95 °C for 5 min. Then, immunoprecipitated samples were separated using SDS–polyacrylamide gels in Tris/Glycine/SDS buffer, transferred onto PVDF membranes, and incubated with anti-HNE4 rabbit polyclonal antibody (abcam, USA, 1:500) overnight at 4 °C. After washing with TBST, the membranes were incubated with anti-rabbit-HRP (1:3000; Cell Signaling Technology) at room temperature for 2 h. Protein bands were detected by ECL Western blotting detection reagents (GE Healthcare). The density of the target protein was quantified using ImageJ program.

## Experiment 2

This experiment was performed to assess the effect of LLL-12 on cerebral blood flow (CBF) deficit of 5XE4 mice. Vehicle and STAT3 inhibitor (LLL-12, 5 mg/kg) were administered daily to 5XE4 mice starting at the age of 6 months. Drug was administered to mice for 2 months. After last dose of drug, CBF was assessed via laser–Doppler probe before and after whisker stimulation.

### Cerebral blood flow during whisker stimulation

Whisker stimulation induced increase in CBF was measured as described previously [28]. Briefly, mice were anesthetized by injecting urethane (750 mg/kg) and chloralose (50 mg/kg) intraperitoneally. After trachea intubation, mice were artificially ventilated. Then, femoral artery was cannulated for recording of arterial blood pressure. Body temperature was maintained at 37 °C using a thermostatically controlled rectal probe connected to a heating lamp. A small craniotomy (2 × 2 mm) was done to expose the barrel cortex, the dura was removed, and the exposed cortex was perfused with Ringer's solution (37 °C; pH 7.3–7.4). CBF was continuously measured at the site of craniotomy with a laser–Doppler probe positioned stereotaxically on the cortical surface. The left vibrissae were cut to a length of 5–10 mm and stimulated for 1 min by gently stroking them (5 Hz) with a cotton-tipped applicator. The right vibrissae were cut as short as possible to avoid unwanted stimulation. Three vibrissal stimulation trials, separated by 10 min intervals, were performed and then, averaged for each experimental group. An experimenter blinded to treatment and surgical groups conducted the analysis.

## Experiment 3

This experiment was performed to assess the effect of LLL-12 on the functional connectivity deficits in 5XE4 mice. Vehicle and STAT3 inhibitor (LLL-12, 5 mg/kg) were administered daily to 5XE4 mice starting at the age of 6 months for 2 months. After last dose of drug, functional connectivity imaging was performed.

### Mounting of glass window

We mounted a glass window over the skull of mice for imaging as described previously [29]. Briefly, mice were anesthetized with 2% isoflurane and shaved their head. Mice were placed on a heating pad maintained at 37 °C and fixed in a stereotactic frame. A midline skin incision was given to expose the skull. The skull was kept moist with an application of mineral oil or artificial cerebrospinal fluid. A glass window (13 mm in diameter and 0.15 mm in thickness) was glued on top of the exposed skull with C&B Metabond Quick Adhesive Cement system, and the skin was glued at the edges of the glass window to close the incision. Then, mice were allowed for recovery for 1 week.

### Functional connectivity optical intrinsic signal (fcOIS) imaging

fcOIS imaging was performed as previously described [30, 31]. Briefly, mice were anesthetized by intraperitoneal injection (i.p.) of a ketamine/xylazine cocktail (ketamine- 86.9 mg/kg; xylazine- 13.4 mg/kg). Depth of anesthesia was measured by respond to hindpaw pinch. A heating pad kept at 37 °C maintained the mouse body temperature. Sequential illumination was provided by 4 light emitting diodes centered at four wavelengths (478 nm, 588 nm, 610 nm, and 625 nm). A cooled, frame-transfer EMCCD camera (iXon 897, Andor Technologies) captured diffuse light reflectance from the skull over a field-of-view of approximately 1 cm^2^. Data were binned 4 × 4 on camera, resulting in a frame rate of 120 Hz (Hemodynamic imaging at 30 Hz). Forty-five minutes of resting state data were collected for each mouse. Raw reflectance images were converted to changes in hemoglobin concentration using the modified Beer–Lambert law.

Analysis of functional connectivity was performed as per our previously published studies [29, 32]. Spontaneous hemodynamic activity was filtered between 0.009 and 0.08 Hz [33] and the time traces of all pixels defined as brain were averaged to create a global brain signal. This global signal was regressed from every pixel's time trace to remove global sources of variance. Regions of interest (seeds) with a diameter of 0.25 mm were placed in functional regions corresponding to cingulate, motor, and somatosensory cortex. Maps of functional connectivity were determined using zero-lag correlation between each region of interest (ROI) and all other time courses in the brain. Maps of global node degree were calculated as described in Hakon et al. [29]. Briefly, the number of functional connections (degree) of each pixel (node) was determined by thresholding whole cortex correlation matrices at z(r) ≥ 0.4, so that only strong positive connections contributed to each pixel’s measure of node degree. Connections above this threshold were set to 1, and summed over both hemispheres to produce a binarized measure of global node degree for each pixel. This procedure was performed in each mouse over pixels within the shared brain mask. The analysis was performed by a researcher who was blinded to treatment and surgical groups.

### Statistical analysis

The statistical analysis was performed using SPSS statistical software package, version 22.0. Results are presented as mean ± SEM. One-way ANOVA was applied to find statistically significant differences among experimental groups. Bonferroni test was used for post-hoc ANOVA analysis. Student’s t-test was also used for statistical comparison. A *p* value < 0.05 was considered as statistically significant.

## Results

### Activated forms of STAT3 are upregulated in cerebral vessels of Alzheimer’s patients and aged 5XE4 mice

**Fig. 1:**
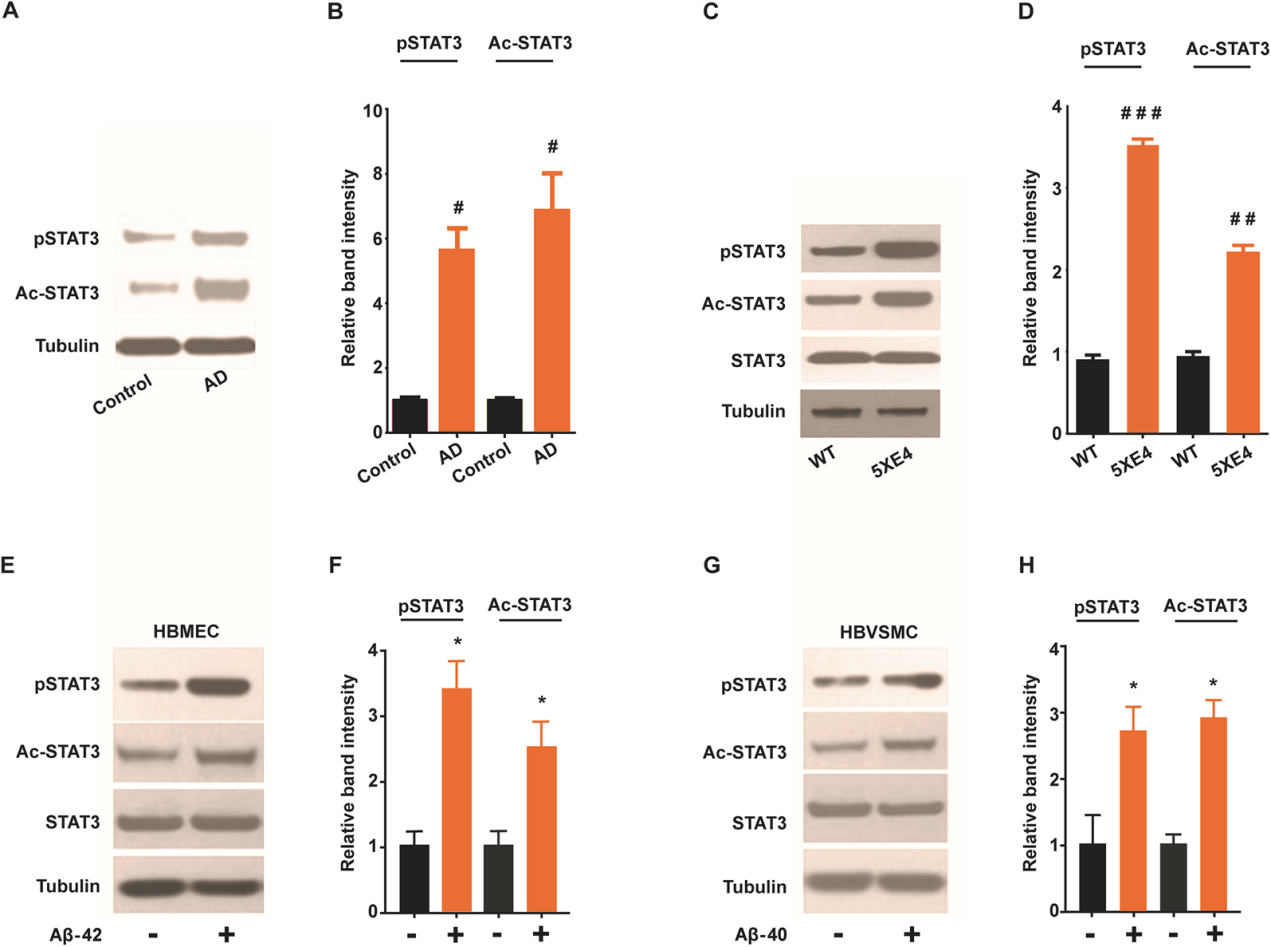
Activated forms of STAT3 is upregulated due to AD-related pathology. **A**, **B** Western blot analysis and quantification of STAT3 expression in blood vessels isolated from brain autopsy samples from age matched control and AD patients. **C**, **D** Immunoblot and quantification of STAT3 expression in blood vessels isolated from brains of 5XE4 and age matched wild-type (WT) mice. **E**, **F** Western blot analysis and quantification of STAT3 expression in cell homogenates following Aβ (1 µM) exposure to human brain microvascular endothelial (HBMEC). **G**, **H** Western blot analysis and quantification of STAT3 expression in cell homogenates following Aβ (1 µM) exposure to human brain vascular smooth muscle cells (HBVSMC). STAT3 phosphorylation at Tyr705 (pSTAT3); STAT3 acetylation at Lys685 (Ac-STAT3). Data is presented as mean ± SEM. #*p* < 0.05, ##*p* < 0.01, ###*p* < 0.001 as compared to control or WT, n = 4–5; *p < 0.05 as compared to no Aβ exposure. The cell culture experiments were performed in triplicate manner

Cerebral blood vessels harboring extensive CAA were isolated from brains of AD patients and 5XE4 mice to study the expression of phosphorylated and acetylated forms of STAT3 (p-STAT3 and ac-STAT3, respectively). Statistical analysis indicated p-STAT3 and ac-STAT3 expression were significantly (*p* < 0.05) upregulated in cerebral blood vessels of AD patients in comparison with age matched controls (Fig. 1B). Additionally, a significant increase in expression of both p-STAT3 (*p* < 0.001) and ac-STAT3 (*p* < 0.01) was noted in cerebral blood vessels of 5XE4 mice when compared with age matched littermate controls (Fig. 1D). These data indicate CAA-laden cerebral vessels develop upregulation of activated forms of STAT3, which suggests a potential role of STAT3 in CAA-induced cerebrovascular dysfunction.

### Activated forms of STAT3 are upregulated in cultured human brain vascular endothelial and smooth muscle cells exposed to Aβ

A previous report indicates that amyloid peptide (Aβ_25–35_) increased expression of phosphorylated and acetylated form of STAT3 in microglia cells. However, there is no report examining the expression of STAT3 in neurovascular compartments exposed to amyloid peptide present in vivo. Therefore, neurovascular cells (HBVSMC and HBMEC) were cultured and incubated with Aβ1-42 (1 µM) and Aβ1-40 (1 µM) for 24 h to study expression of activated forms of STAT3. Figure 1E–H represent p-STAT3 and ac-STAT3 expression in HBMEC and HBVSMC. The results indicate Aβ42 (1 µM) significantly (*p* < 0.05) enhances the expression of p-STAT3 and Ac-STAT3 in HBMEC (Fig. 1E, F). Furthermore, Aβ40 (1 µM) also significantly (*p* < 0.05) increases the expression of phosphorylated and acetylated form of STAT3 in HBVSMC (Fig. 1G, H). Taken together, these results show that exposure of human cerebral vascular cells (HBMEC and HBVSMC) to Aβ leads to increased expression of activated forms of STAT3.

### STAT3 inhibition reduces Aβ-induced oxidative stress in human brain vascular smooth muscle and vascular endothelial cells

**Fig. 2:**
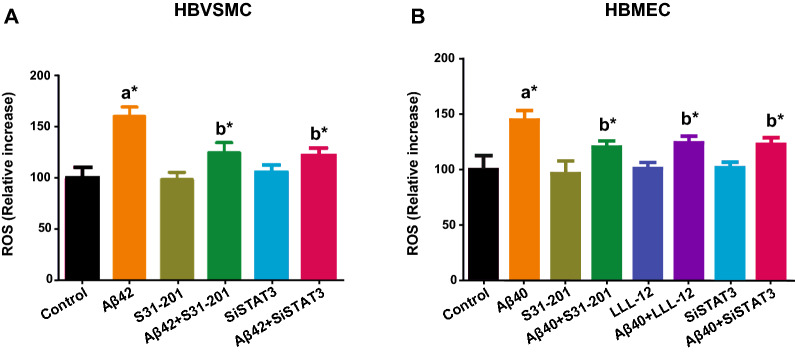
Pharmacological and genetic inhibition of STAT3 attenuates Aβ induced oxidative stress. **A** Human vascular smooth muscle cells (HBVSMC) was pretreated with vehicle or STAT3 inhibitor S31-201 (10 μM) or siSTAT3. **B** Human brain microvascular endothelial cells (HBMEC) was pretreated with vehicle or STAT3 inhibitor S31-201 (10 μM) or LLL-12 (5 μM) or siSTAT3. Cells were treated with Aβ (1 µM) and oxidative stress was measured by increase in dihydroethidium (DHE) fluorescence. Each experiment was run in triplicate manner. Data is presented as mean ± SEM. *p < 0.05, a- as compared to control; b- as compared to Aβ40 and Aβ42

Several studies indicate oxidative stress develops in the early stages of AD [34, 35], that various Aβ species cause oxidative stress especially in cerebral vascular cells [18, 36, 37], and that a key source of this Aβ-induced oxidative stress is NADPH oxidase [36]. However, the upstream molecular mechanisms by which Aβ produces NADPH oxidase-mediated oxidative stress are poorly understood. Because STAT3 has recently been implicated in angiotensin-induced vascular oxidative stress [38], we examined whether STAT3 mediates Aβ-induced vascular oxidative stress in cultured cells. Specifically, we exposed cultured HBVSMC and HBMEC to Aβ42 and Aβ40 (1 µM), respectively and found that Aβ42 significantly (*p* < 0.05) increased generation of ROS in HBVSMC as compared to control treatment (Fig. 2A), and that pharmacological inhibition of STAT3 via S31-201 treatment significantly (*p* < 0.05) blocked this ROS response (Fig. 2A). Similarly, genetic knockdown of STAT3 via siRNA treatment was found to significantly (*p* < 0.05) block Aβ42-induced ROS production in HBVSMC (Fig. 2A). These findings were also confirmed and replicated in human brain vascular endothelial cells using Aβ40 as the ROS generator. Specifically, we found that Aβ40 exposure to HBMEC significantly (*p* < 0.05) increases ROS production as compared to control (Fig. 2B), and that pharmacological inhibition of STAT3 via S31-201 significantly (*p* < 0.05) blocked this ROS response (Fig. 2B). These results were further confirmed using another STAT3 inhibitor, LLL-12 (Fig. 2B). Similarly, genetic inhibition of STAT3 via siRNA treatment was found to significantly (*p* < 0.05) block Aβ40-induced ROS production in HBMEC (Fig. 2B). Taken together, these data indicate STAT3 plays a key causal role in the oxidative stress produced by exposure of cultured vascular cells to Aβ40 and Aβ42 species.

### STAT3 inhibition improves cognitive function of aged 5XE4 mice

**Fig. 3:**
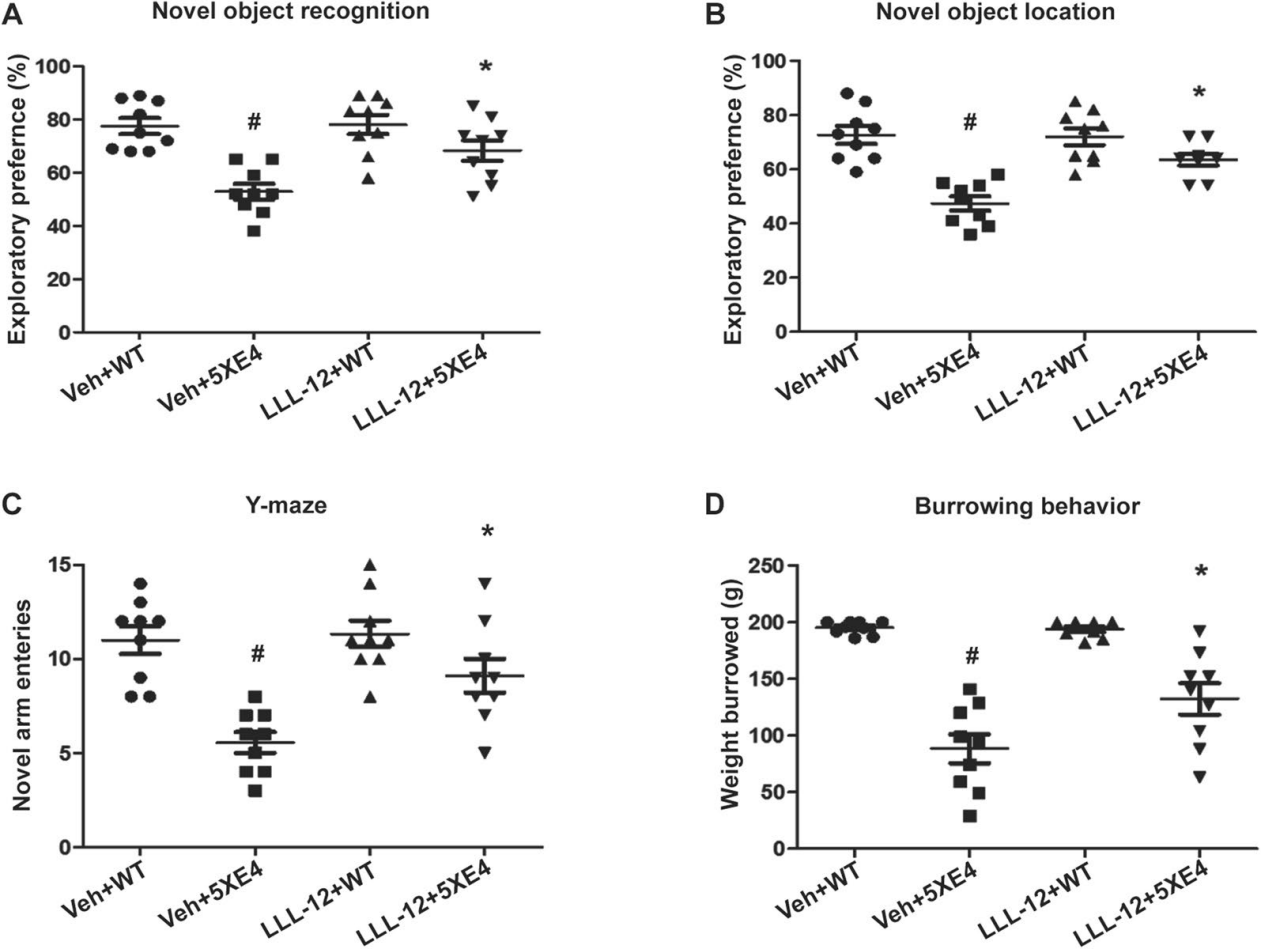
STAT3 inhibition rescues the behavioral deficits of 5XE4 mouse model of AD. The cognitive function in experimental mice was assessed by measuring **A** exploratory preference in novel object recognition test, **B** exploratory preference in novel object location task, **C** novel arm entries in Y-maze test, **D** weight burrowed in burrowing test. Data is presented as mean ± SEM, #,*p < 0.05, #- as compared to veh + WT, *- as compared to veh + 5XE4, n = 9–10 in each group, veh- vehicle, WT- wild-type, 5XE4- 5XFAD/APOE4

To explore the role of STAT3 in amyloid-induced neurovascular dysfunction in vivo, 6-month-old 5XE4 mice were treated with the STAT3-specific inhibitor, LLL-12, or vehicle for 2 months. Several neurobehavioral paradigms were then performed to assess memory function of experimental mice including object recognition, y-maze, and burrowing tests. In novel object recognition (NOR) test that assesses prefrontal cortex-dependent memory and novel object location (NOL) test that assesses hippocampal-dependent memory, we found that the exploratory preferences of vehicle-treated 5XE4 mice were significantly (*p* < 0.05) decreased compared to vehicle-treated wild-type mice (Fig. 3A, B). Inversely, aged 5XE4 mice treated with LLL-12 had a significant (*p* < 0.05) increase in exploratory preferences in NOR and NOL tests as compared to vehicle-treated aged 5XE4 mice (Fig. 3A, B). No significant differences in NOR and NOL tests were found in wild-type mice treated with LLL-12 vs. vehicle (Fig. 3A, B).

In the Y-maze test that assesses hippocampal-dependent memory, vehicle-treated aged 5XE4 mice had a significant decrease in novel arm visits as compared to vehicle-treated wild-type mice (Fig. 3C). Conversely, LLL-12 treatment led to a significant (*p* < 0.05) increase in novel arm entries in aged 5XE4 mice as compared to aged vehicle-treated 5XE4 mice (Fig. 3C). No significant difference in novel arm visits was found between LLL-12 and vehicle treated wild-type mice (Fig. 3C).

In the burrowing test, vehicle-treated aged 5XE4 mice had a significant (*p* < 0.05) reduction in weight burrowed as compared to vehicle-treated wild-type mice (Fig. 3D). In contrast, LLL-12 treatment led to a significant (*p* < 0.05) increase in weight burrowed by aged 5XE4 mice as compared to vehicle-treated aged 5XE4 mice (Fig. 3D). No significant difference in weight burrowed was found in LLL-12 and vehicle treated wild-type mice (Fig. 3D).

Taken together, these data indicate pharmacologic inhibition of STAT3 via LLL-12 administration markedly reduces memory dysfunction in aged 5XE4 mice without a negative effect of this pharmacotherapy on memory of healthy mice. These results highlight the promising therapeutic potential of STAT3 inhibition for amyloid-induced dementia and raise questions regarding the role of STAT3 in amyloid pathogenesis.

### STAT3 inhibition reduces CAA, brain parenchymal amyloid plaque load, soluble and insoluble Aβ levels via attenuation of APP processing in brains of aged 5XE4 mice

**Fig. 4:**
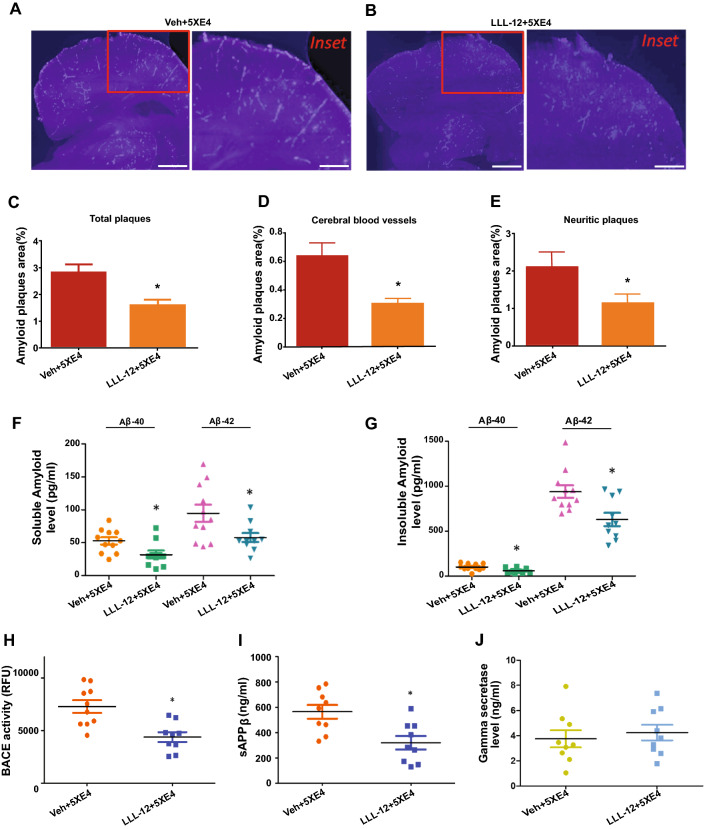
STAT3 inhibition reduces AD-related pathologies in 5XE4 mouse model of AD. STAT3 inhibitor treatment for 2 months decreases CAA and parenchymal plaques load. **A**, **B** Photomicrographs of mouse brain sections stained with methoxy-X34 to determine the amyloid plaques in brain and blood vessels. **C** Total amyloid load in 5XE4 mice brain. **D** CAA load in 5XE4 mice brain. **E** Neuritic/parenchymal plaques burden in 5XE4 mice brain. Scale bars represent 500 mm for section and 100 mm for inset section. STAT3 inhibition also reduces soluble and insoluble amyloid fractions in 5XE4 mouse model of AD. **F** Soluble Aβ levels in AD mice brain. **G** Insoluble Aβ levels in AD mice brain. Reduced amyloid processing by STAT3 inhibitor in 5XE4 mice was determined by measuring, **H** BACE1 activity, **I** sAPPβ levels, **J** γ-secreatse level. Treatment with STAT3 inhibitor deceased BACE1 activity and sAPPβ levels in brain of 5XE4 mice without affecting γ-secreatse level. Data is presented as mean ± SEM, *p < 0.05 as compared to veh + 5XE4, n = 9–11 in each group, veh- vehicle, 5XE4- 5XFAD/APOE4

To begin to explore the underlying mechanisms by which STAT3 inhibition improves the neurobehavioral performance of aged 5XE4 mice, we examined amyloid plaque loads from aged 5XE4 mice that had been treated with LLL-12 vs. vehicle. Figure 4A, B show the distribution of neuritic plaques and vascular amyloid load in LLL-12 vs. vehicle treated aged 5XE4 mice. Our results show that aged 5XE4 mice treated with LLL-12 experienced a significant (*p* < 0.05) reduction in total amyloid load, neuritic plaque load, and CAA load as compared to vehicle-treated aged 5XE4 mice (Fig. 4C–E). These results indicate that one way by which STAT3 inhibition may be improving neurobehavioral deficits in aged 5XE4 mice is through a reduction in parenchymal and vascular amyloid deposition.

To further delineate the impact of STAT3 inhibition on Aβ deposition and to begin to elucidate the mechanisms by which STAT3 inhibition positively impacts amyloid deposition, we biochemically assessed soluble and insoluble levels of amyloid and examined BACE1 activity and γ-secretase levels in brain homogenates of aged 5XE4 mice treated with LLL-12 vs. vehicle. We found that LLL-12 significantly (*p* < 0.05) reduced soluble and insoluble Aβ40 and Aβ42 levels in aged 5XE4 mice brain as compared to vehicle-treated aged 5XE4 mice (Fig. 4F, G). We also found that APP processing was attenuated, as evidenced by a significant reduction in BACE1 activity and soluble APPβ levels (Fig. 4H, I). We did not find an impact of LLL-12 treatment on γ-secretase levels (Fig. 4J). Taken together, these results suggest STAT3 inhibition decrease soluble- and insoluble- Aβ40 and Aβ42 levels in aged 5XE4 mice at least in part by mitigating APP processing through a reduction in BACE1 activity.

### STAT3 inhibition reduces oxidative stress, attenuates oxidized LRP-1 levels, and improves LRP-1 levels in aged 5XE4 mice

**Fig. 5:**
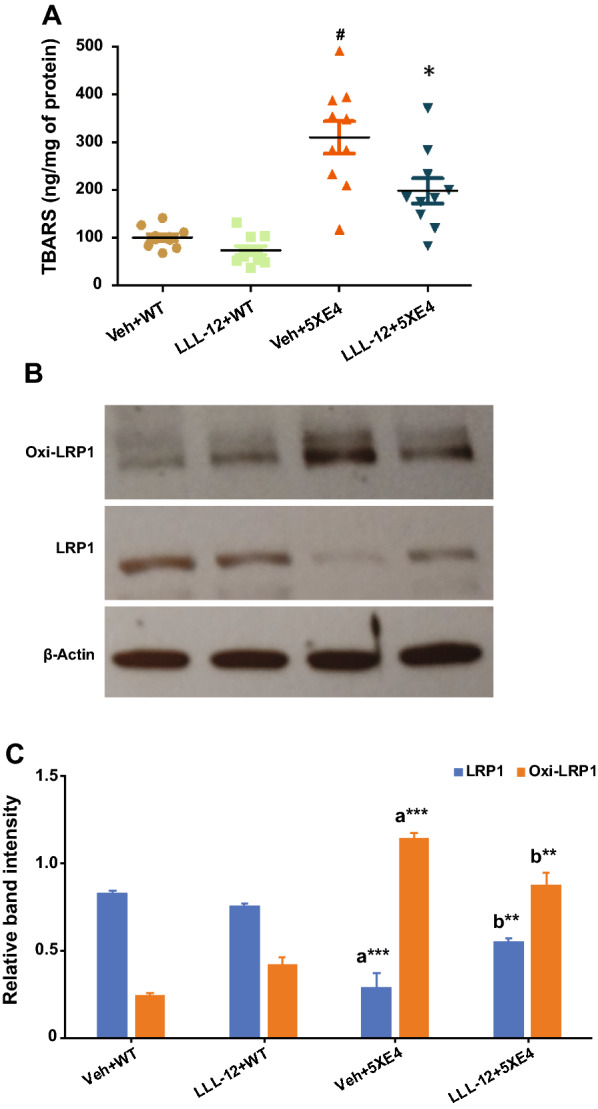
STAT3 inhibition reduces oxidative stress and oxidative modification of LRP-1 and restores LRP-1 level in 5XE4 mouse model of AD. **A** Oxidative stress was assessed by measuring TBARS levels in mice brain. Data is presented as mean ± SEM. #*p* < 0.05 as compared to veh + WT, *p < 0.05 as compared to veh + 5XE4; n = 9–10. LRP-1 changes in mice brain homogenate were assessed through, **B** western blot for LRP-1 and oxi-LRP-1 expression; **C** quantification of expression LRP-1 and oxi-LRP-1. Data is presented as mean ± SEM. *p < 0.05, **p < 0.01, ***p < 0.001, a- as compared to veh + WT, b- as compared to veh + 5XE4; n = 4 for western blot analysis; veh- vehicle, WT- wild-type, 5XE4- 5XFAD/APOE4

To explore additional mechanisms by which STAT3 inhibition positively impacts amyloid deposition, we further examined whether LLL-12 treatment reduces amyloid-induced oxidative stress and its impact on LRP-1—a key molecule involved with Aβ clearance across the blood–brain barrier (BBB). To assess oxidative stress, a TBARS assay was used to measure lipid peroxidation in brains of aged 5XE4 mice. We found that TBARS levels were significantly increased (*p* < 0.05) in aged 5XE4 mice treated with vehicle as compared to age-matched wild-type mice (Fig. 5A). We also found that LLL-12 treatment significantly attenuated (*p* < 0.05) the increase in TBARS levels found in aged 5XE4 mice in comparison with vehicle treated 5XE4 mice (Fig. 5A). No impact of LLL-12 treatment on TBARS levels was noted in wild-type mice (Fig. 5A). In total, these data suggest STAT3 plays a key role in the amyloid-induced oxidative stress seen in aged 5XE4 mice.

Next, we examined the potential impact of STAT3-mediated oxidative stress on LRP-1, which has been previously implicated in Aβ clearance and AD pathophysiology [39, 40]. In particular, we examined levels of LRP-1 and oxidized LRP-1—the latter being an altered form of this key Aβ clearance molecule that has been identified in AD patients [41]. To assess the oxidized modification of LRP-1, we measured expression of HNE-bound LRP-1 (oxi-LRP-1). Our results indicate LRP-1 levels were significantly decreased (*p* < 0.001) and oxi-LRP-1 levels were significantly increased (*p* < 0.001) in brain of aged 5XE4 mice as compared to aged-matched wild-type mice (Fig. 5C). However, LLL-12 treatment was found to significantly increase LRP-1 levels (*p* < 0.01) and significantly decrease oxi-LRP-1 levels (*p* < 0.01) in brain of in aged 5XE4 mice (Fig. 5C). No significant change in LRP-1 and oxi-LRP-1 levels were found between vehicle and LLL-12 treated wild-type mice (Fig. 5C). In total, these data suggest that one consequence of STAT3-mediated oxidative stress in aged 5XE4 mice appears to be degradation of LRP-1 and reduced overall LRP-1 levels, which would negatively impact Aβ clearance at the BBB.

### STAT3 inhibition rescues neuroinflammation in aged 5XE4 mice

**Fig. 6:**
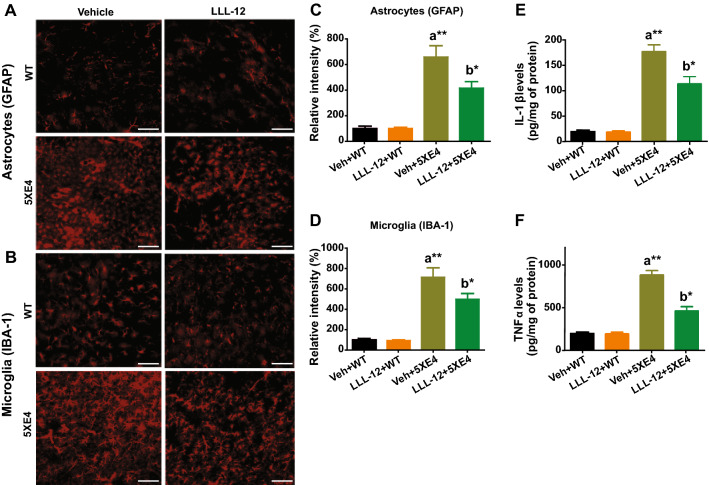
STAT3 inhibition reduces neuroinflammation in 5XE4 mouse model of AD. The mouse brain sections were immunostained with IBA-1 and GFAP for microgliosis and astrocytosis, respectively. Proinflammatory cytokines (IL-1β and TNFα) levels in mice brain were also measured using appropriate ELISA kits. **A**, **B** Photomicrographs of immunostaining of GFAP (a marker of astrocyte) and IBA-1 (a marker for microglia). **C**, **D** Quantification of relative intensity of GFAP and IBA-1. Scale bars represent 100 mm for brain section. **E** IL-1β levels in mice brain. **F** TNFα levels in mice brain. Data is presented as mean ± SEM, *p < 0.05, **p < 0.01, a- as compared to veh + WT, b- as compared to veh + 5XE4, n = 9–10 in each group, veh- vehicle, WT- wild-type, 5XE4- 5XFAD/APOE4

The role of neuroinflammation in AD pathophysiology has been well described [42], including increased expression of activated microglia and astrocytes and high levels of cytokines in AD mouse models and patients [42]. Therefore, we assessed astrocytosis, microgliosis and cytokines levels to measure neuroinflammation in aged 5XE4 mice treated with LLL-12 or vehicle. Our results show astrocytosis via GFAP staining and microgliosis via IBA-1 staining were significantly increased (*p* < 0.01) in aged 5XE4 mice as compared to age-matched wild-type mice (Fig. 6C, D). In aged 5XE4 mice treated with LLL-12, however, this GFAP and IBA-1 immunoreactivities were significantly reduced (*p* < 0.05) compared to vehicle treatment (Fig. 6C, D). No significant differences were found in GFAP and IBA-1 immunostaining in wild-type mice treated with LLL-12 vs. vehicle (Fig. 6C, D). In addition, we found a significant increase (*p* < 0.01) in IL-1β and TNFα levels in brain of aged 5XE4 mice vs. age-matched wild-type mice (Fig. 6E, F). In aged 5XE4 mice treated with LLL-12, however, this augmentation in inflammatory cytokines was significantly reduced (*p* < 0.05) compared to vehicle treatment (Fig. 6E, F). No significant differences were found in IL-1β and TNFα levels in brain in wild-type mice treated with LLL-12 vs. vehicle (Fig. 6E, F). In total, these results indicate aged 5XE4 mice develop marked neuroinflammation that can be attenuated by pharmacologic STAT3 inhibition.

### STAT3 inhibition improves whisker stimulation-evoked CBF and enhance functional brain organization in aged 5XE4 mice

**Fig. 7:**
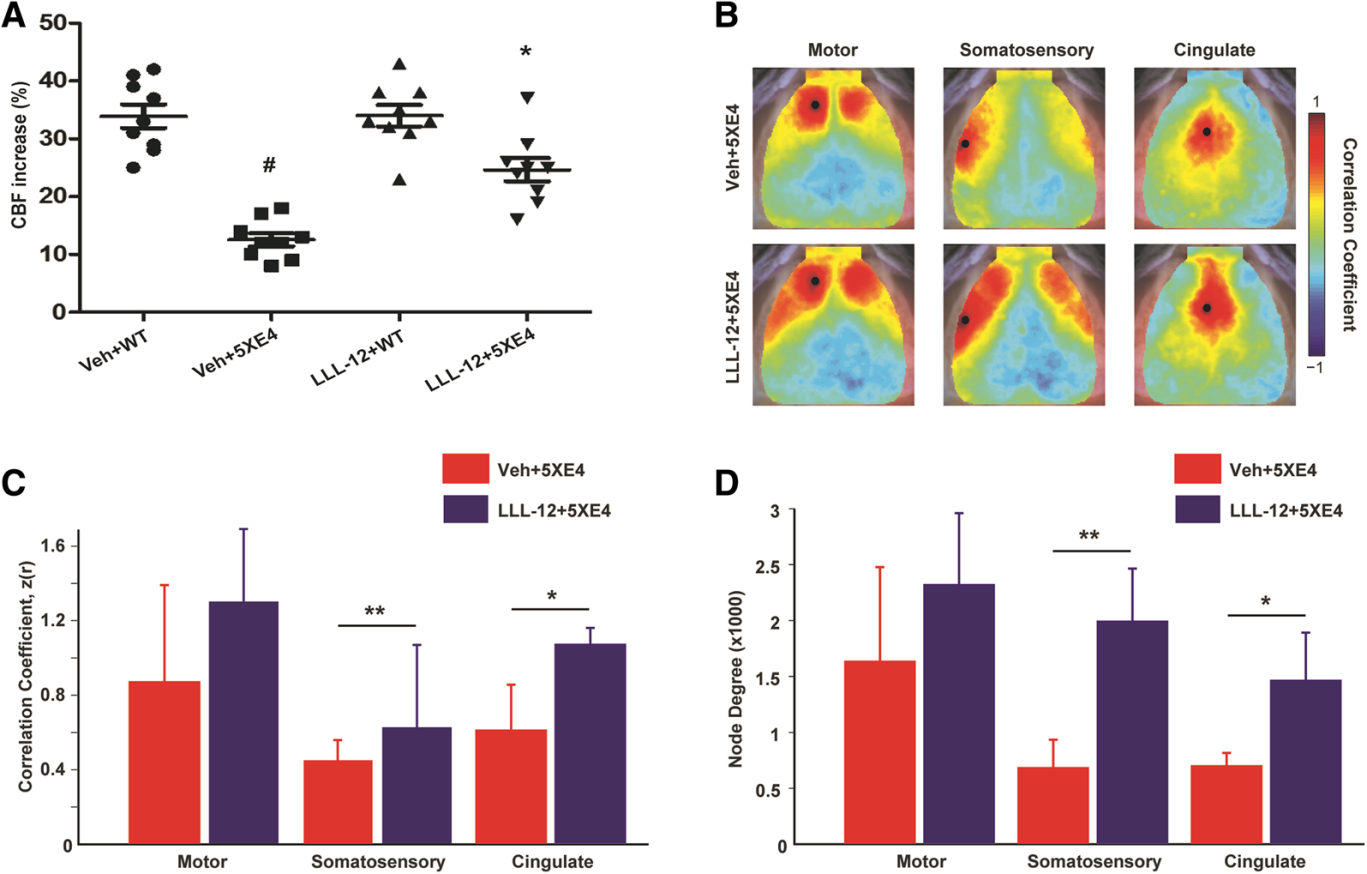
STAT3 inhibition improves functional CBF and brain functional connectivity (FC) in 5XE4 mouse model of AD. **A** Mice underwent somatosensory cortex CBF measurement via laser-Doppler flowmetry (% change after whisker stimulation). Functional connectivity was measured via functional connectivity optical intrinsic signal imaging. **B** Maps of FC in motor, somatosensory and cingulate cortices of both groups. Notice increased positive correlation magnitude (reds) in LLL-12 treated animals. **C** Quantification of homotopic (bilateral) functional connectivity reveals significantly increased FC somatosensory and cingulate cortices following STAT3 inhibition. **D** The number of functional connections in each group displayed as node degree (see methods). In the LLL-12 treated group, both somatosensory and cingulate cortices exhibited more positive correlations across both hemispheres compared to aged 5XE4 mice. Data is presented as mean ± SEM. #,*p < 0.05, **p < 0.01, #- as compared to veh + WT, *- as compared to veh + 5XE4; n = 9–10 in each group for CBF experiment; n = 5 in each group for functional connectivity experiment, veh- vehicle, WT- wild-type

We measured whisker stimulation-induced CBF in barrel cortex of aged 5XE4 mice treated with LLL-12 or vehicle. A significant decrease (*p* < 0.05) in whisker-evoked CBF was found in aged 5XE4 mice compared to age-matched wild-type (Fig. 7A). However, LLL-12 treatment significantly improved (*p* < 0.05) whisker-induced CBF in aged 5XE4 mice compared to vehicle treatment (Fig. 7A). No significant change in whisker-evoked CBF was noted in wild-type mice treated with LLL-12 vs. vehicle (Fig. 7A). These results show that the alterations in CBF responses found in aged 5XE4 mice can be restored via pharmacologic STAT3 inhibition.

Previously, we have shown that fcOIS is sensitive to functional disruption in APP/PS1 mice after Aβ deposition [43], and that changes in neural network function following treatment with anti-apoE antibodies are associated with reduced neuroinflammation [44]. Given the effects of STAT3 inhibition on stimulus evoked CBF and gliosis, we next sought to evaluate whether LLL-12 treatment altered patterns of spontaneous hemodynamic activity across resting state networks in aged 5XE4 mice (Fig. 7B–D). Qualitatively, mice given LLL-12 treatment exhibit patterns of functional connectivity associated with healthier brain function. For example, maps of regional functional connectivity in the LLL-12 treated group reveal higher correlation strength (reds) ipsilateral to the seed, as well as across hemispheres (Fig. 7B, C). Vehicle treated 5XE4 mice exhibited significantly reduced functional connectivity strength between homotopic somatosensory (*p* < 0.01) and cingulate (*p* < 0.05) cortical areas compared to those treated with LLL-12 (Fig. 7B, C). To further characterize map differences, the number of functional connections (pixels having a correlation coefficient z(r) > 0.4)) was quantified in both groups. Significantly higher node degree was observed in aged 5XE4 mice treated with LLL-12 within somatosensory (*p* < 0.01) and cingulate (*p* < 0.05) cortices compared to vehicle (Fig. 7D). Together, these results demonstrate that STAT3 inhibition improves functional brain organization of aged 5XE4 mice.

## Discussion

Our study has several important findings. First, broad inhibition of STAT3 via administration of the STAT3-specific inhibitor, LLL-12, markedly improved multiple histological, biochemical, physiological, and functional endpoints in a mouse model of amyloidosis. Specifically, broad pharmacologic inhibition of STAT3 was found to reduce parenchymal and vascular amyloid deposits, attenuate soluble and insoluble brain Aβ levels, decrease neuroinflammation, inhibit oxidative stress, and augment neurobehavioral function in aged 5XE4 mice. Second, an important role of STAT3 in Aβ-induced cerebrovascular pathology was identified. Specifically, we found that 1) Aβ species applied to cultured vascular cells increase activated forms of STAT3 and produce oxidative stress in a STAT3-dependent manner; 2) CAA-laden cerebral vessels from aged 5XE4 mice and autopsied AD patients have markedly increased levels of activated STAT3; and 3) pharmacologic inhibition of STAT3 reduces CAA load, improves cerebral hemodynamics, and restores functional network communication in aged 5XE4 mice. Third, two potential mechanisms accounting for the multifaceted beneficial effects of broad STAT3 inhibition in aged 5XE4 mice were identified: (1) reduction in BACE1 activity and APP processing; and (2) attenuation of LRP-1 oxidation leading to restoration of brain levels of LRP-1. Taken together, these results provide key validation of the recent findings by Reichenbeach and colleagues [12] who showed that astrocyte-specific deletion of STAT3 reduces neuroinflammation, neuritic plaque deposition, and neurobehavioral deficits in aged APP/PS1 mice. Importantly, our results advance upon these initial observations in three significant ways. First, we demonstrate that broad STAT3 inhibition (i.e. beyond astrocyte-specific gene deletion) provides robust protection not only against neuritic plaques and neuroinflammation, but also provides marked protection against vascular amyloid in the form of CAA and cerebrovascular dysfunction. Second, we showed that the protection afforded by STAT3 inhibition goes well beyond astrogliosis, including strong beneficial effects on oxidative stress, cerebral hemodynamics, and functional connectivity. Third, we identified two molecular mechanisms by which STAT3 inhibition may directly impact parenchymal and vascular amyloid pathogenesis—reduction in BACE1 activity and restoration of brain levels of LRP-1.

### STAT3 inhibition provides multifaceted neurovascular protection in aged 5XE4 mice

STAT3, a member of the STAT family, is a transcription factor with well-described roles in various biological processes including cell proliferation, differentiation, and death. It is activated through JAK-mediated phosphorylation in response to endogenous substances including numerous cytokines [7–9], and through other post-translational modifications such as acetylation [10]. A central role of JAK/STAT3 signaling in astrogliosis is well documented in several neurological conditions including stroke [13], AD [11, 12], spinal cord injury [45, 46], multiple sclerosis [47], and epilepsy [14]. In addition, a causal role of STAT3 in amyloid-induced neuroinflammation and neuritic plaque formation has recently been identified [12]. While the beneficial effects of drugs that non-specifically inhibit STAT3 have been reported in transgenic mouse models of amyloidosis [48, 49], direct evidence that STAT3-specific pharmacologic inhibitor attenuates amyloid deposition and ameliorates amyloid-induced neurovascular dysfunction is lacking. Therefore, in the present study, we sought to answer these important questions, and also begin to explore the underlying molecular mechanisms by which STAT3-mediated amyloid and neurovascular protection occurs.

Several groups have reported that Aβ peptides in vitro or fibrillar amyloid pathology in vivo are associated with increased levels of activated STAT3 [12, 50], however, the potential for STAT3 to play a causal role in amyloid formation and amyloid-induced neural dysfunction has only recently been examined. First, Wan and colleagues noted that Aβ exposure to cultured rat neurons or intrahippocampal injection in mice induces tyrosine phosphorylation of STAT3 in neurons, and that genetic and pharmacologic inhibition of STAT3 protected cultured cortical neurons from Aβ-induced apoptosis [50]. Second, Reichenbach and colleagues reported that astrocyte-specific genetic knockout of STAT3 in aged APP/PS1 mice reduced Aβ levels and neuritic plaque load, decreased pro-inflammatory cytokine activation and dystrophic neurite burden, and improved spatial learning and memory [12]. These investigators, however, did not see a reduction in neuritic plaque load in APP/PS1 mice treated with the STAT3 inhibitor, SH-4–54, although improvements in peri-plaque astrocyte activation and neurobehavioral outcome were noted [12]. Importantly, neither of these studies examined the potential role of STAT3 in the profound cerebrovascular deficits found in mouse models of amyloidosis and AD patients.

In our study, we found that broad STAT3 inhibition via 8-week administration of the STAT3-specific inhibitor, LLL-12, provided multifaceted neural and vascular protection in aged 5XE4 mice, leading to significant improvements in neurobehavioral outcome as measured by several well-validated cognitive assessments. This neurovascular protection included reduction in both neuritic plaque and CAA load, decreased brain levels of soluble and insoluble Aβ, reduced neuroinflammation, less oxidative stress, improvements in focal CBF responses following whisker-stimulation, and restored resting-state functional connectivity. In total, our results indicate STAT3 inhibition not only protects against amyloid pathology and neurobehavioral dysfunction through its impact on astrogliosis [12], but that STAT3 inhibition provides broader levels of neurological protection through other cell types and mechanisms including those associated with the neurovascular unit.

### STAT3 inhibition reduces vascular oxidative stress and CAA deposition, improves cerebral hemodynamics, and augments functional connectivity

We provide several lines of evidence that STAT3 plays a causal role in the vascular pathology and cerebrovascular deficits associated with AD. First, we show that Aβ species applied to cultured vascular cells increase activated forms of STAT3 and produce significant vascular oxidative stress in a STAT3-dependent manner. We also show CAA-laden vessels from aged 5XE4 mice and AD patients have increased levels of activated forms of STAT3. These results expand upon past studies that show 1) Aβ species increase STAT3 activation in cultured neurons and microglia, and 2) amyloid pathology in AD mice and AD patients is associated with increased levels of activated STAT3 [11, 50–52]. Second, we found that broad pharmacologic inhibition of STAT3 via LLL-12 administration markedly reduces CAA and improves stimulus evoked cerebral perfusion in aged 5XE4 mice. The latter is of particular importance given that both of these measures—CAA load and diminished cerebral hemodynamics—have been linked to the onset and severity of neurobehavioral deficits in mouse models of amyloidosis [53] and the cognitive dysfunction of AD patients [54, 55]. Third, we found that LLL-12 treatment of aged 5XE4 mice restored functional communication within sensorimotor and cingulate networks. Spontaneous resting-state fluctuations represent highly organized synaptic activity within the brain [56]. Coupling between changes in cerebral oxygenation (i.e., fcMRI and fcOIS imaging) and slow (< 0.1 Hz) fluctuations in local field potentials have been established across species [57–59]. Importantly, functional connectivity disruption is an important biomarker of neurological disease [60], while restoration of functional connectivity within and across networks correlates with behavioral recovery [44, 61]. The finding that LLL-12 treatment restores both local (task-evoked CBR responses) and global (resting state FC) hemodynamic activity suggests more intact neural network integrity and neurovascular coupling in aged 5XE4 mice.

In total, our in vitro and in vivo data implicating STAT3 in CAA pathogenesis and CAA-induced cerebrovascular deficits represent a significant advancement, as they strongly suggest STAT3 not only plays a causal role in the parenchymal pathology and Aβ-related deficits of AD, but that it also contributes to the vascular pathology and dysfunction associated with AD-related dementia. Moreover, our results indicate pharmacologic inhibition of STAT3 is a promising therapeutic approach for patients with AD and/or CAA deserving of additional investigation.

### Two potential mechanisms by which STAT3 inhibition attenuates amyloid deposition

In a past in vitro study by Liu and colleagues [62], evidence was provided suggesting STAT3 may play a role in AD pathophysiology via its role in regulating expression levels of BACE1 and the catalytic subunit of γ-secretase, presenilin-1—molecules with well-characterized roles in catalyzing APP cleavage into Aβ fragments and promoting amyloid deposition. In vivo data supporting this observation, however, has yet to be reported. In the present study, treatment with the STAT3 inhibitor, LLL-12, caused a significant decrease in neuritic plaque and CAA formation and a marked reduction in soluble and insoluble Aβ levels in brains of aged 5XE4 mice. To determine whether this potential regulatory role of STAT3 in BACE1 and γ-secretase expression underlies the observed impact of STAT3 inhibition on Aβ levels and amyloid pathogenesis, we examined BACE1 activity, levels of γ-secretase and soluble APPβ. Though no effect on γ-secretase activity was noted, we did find a reduction in BACE1 activity and a corresponding decrease in soluble APPβ levels in LLL-12-treated 5XE4 mice. When combined with results from the in vitro study by Liu and colleagues [62], our in vivo results suggest one mechanism by which STAT3 inhibition attenuates amyloid pathogenesis and its associated neurovascular deficits is by attenuating APP processing through inhibition of BACE1 activity.

Another potential mechanism by which STAT3 contributes to the pathogenesis of AD and CAA is through an upstream regulatory role in Aβ-induced production of ROS. We [5, 18] and others [36, 37, 63, 64] have previously provided multiple levels of evidence that ROS play an important role in the neurovascular deficits of AD and CAA, and in the pathogenesis of CAA itself. The molecular cascades by which Aβ species produce this oxidative stress, however, are incompletely understood—although NADPH oxidase appears to be a critical downstream molecular producer of Aβ-induced oxidative stress [36, 37, 63, 64]. Outside the AD literature, a growing body evidence indicates STAT3 plays an important role in oxidative stress and its impact on cellular processes and disease. For example, oxidative stress activates STAT3 via inhibition of protein-tyrosine phosphatase activity leading to enhanced phosphorylation and nuclear translocation of this transcription factor [65, 66]. STAT3 has also been implicated as an upstream inducer of oxidative stress. For example, Faraci and colleagues demonstrated the involvement of STAT3 in angiotensin-induced vascular oxidative stress, endothelial dysfunction and hypertension and these effects were prevented by STAT3 inhibitor [38]. Taken together, these data suggest that a reciprocal regulatory relationship between STAT3 and oxidative stress exists. When applied to AD and CAA, these data raise the intriguing possibility that STAT3-mediated production of ROS could contribute to amyloid pathogenesis and amyloid-induced neurovascular deficits.

In the present study, we demonstrated that pharmacological inhibitors of STAT3 (S31-201 and LLL-12) ameliorated Aβ-induced generation of ROS in cultured vascular smooth muscle cells and endothelial cells. We also found that genetic inhibition of STAT3 via siRNA treatment similarly attenuated Aβ-induced generation of ROS in these cell lines. These in vitro data support the notion that STAT3 is an important inducer of oxidative stress following exposure to Aβ species. To further investigate this possibility, we examined in vivo whether STAT3 inhibition impacts oxidative stress in brain of aged 5XE4 mice. We found that levels of TBARS, an oxidative product from lipid, was markedly increased in aged 5XE4 mice, which is consistent with past reports of enhanced reactive oxygen species in mouse models of amyloidosis [5, 37]. Importantly, we found that this oxidative stress was significantly reduced in aged 5XE4 mice treated with the STAT3 inhibitor, LLL-12. Next, we examined whether the impact of STAT3 inhibition on oxidative stress levels alters expression levels of LRP-1—a molecule that plays an important role in regulating Aβ clearance including mediating Aβ efflux across the blood–brain barrier [67]. We found that oxidized LRP-1 levels were increased and non-oxidized LRP-1 levels were decreased in aged 5XE4 mice. We also found that treatment with the STAT3-specific inhibitor, LLL-12, reversed these pathologic alterations in LRP-1 expression in aged 5XE4 mice. Taken together, our in vitro and in vivo results suggest that another potential mechanism by which STAT3 inhibition attenuates amyloid pathogenesis and its associated neurovascular deficits is by reducing Aβ-induced oxidative stress production and its pathologic impact on LRP-1 expression.

## Conclusion

Findings from the present study provide multiple in vitro and in vivo lines of evidence that STAT3 has an important contribution to the pathogenesis of both parenchymal and vascular amyloid deposits and the neurovascular deficits that they cause. These results are not only an important validation of the recent results from Reichenbach and colleagues who showed that astrocyte‐specific deletion of STAT3 reduced neuritic plaque load, decreased dystrophic neurite burden, and improved spatial learning and memory in aged APP/PS1 mice [12]; they expand upon this initial observation in several important ways. Specifically, we showed that STAT3 plays a broader, multifaceted role in AD pathophysiology including a contribution to vascular oxidative stress, CAA pathogenesis, cerebral autoregulatory dysfunction, and functional connectivity deficits. Our results also begin to shed light on the molecular underpinnings by which STAT3 contributes to AD and CAA pathology, including providing evidence that STAT3 (1) impacts APP processing through BACE1 activity, and (2) induces oxidative stress that alters LRP-1 expression. In total, our results indicate STAT3 has an important, multifaceted role in the pathophysiology of AD and CAA and that therapeutic strategies to inhibit STAT3 carry great promise for patients suffering from these conditions.

## Data Availability

The datasets used and/or analyzed during the current study are available from the corresponding author on reasonable request.

## Abbreviations

Aβ: Amyloid-β
ACSF: Artificial cerebrospinal fluid
AD: Alzheimer’s disease
ApoE: Apolipoprotein E
APP: Amyloid precursor protein
CAA: Cerebral amyloid angiopathy
CBF: Cerebral blood flow
CSF: Cerebrospinal fluid
FcOIS: Functional connectivity optical intrinsic signal
GFAP: Glial fibrillary acidic protein
HBMEC: Human brain microvascular endothelial cells
HBVSMC: Human brain vascular smooth muscle cells
JAK: Janus kinases
LRP-1: Lipoprotein receptor related protein-1
MDA: Malondialdehyde
PBS: Phosphate-buffered saline
PFA: Paraformaldehyde
ROS: Reactive oxygen species
SDS: Sodium dodecyl sulphate
SOCS3: Suppressor of cytokine signaling 3
STAT3: Signal transducer and activator of transcription 3;
TBARS: Thiobarbituric acid reactive species
TBST: Tris buffered saline with Tween
WT: Wild-type
